# Treatment With Nepicastat Decreases Contextual Traumatic Memories Persistence in Post-traumatic Stress Disorder

**DOI:** 10.3389/fnmol.2021.745219

**Published:** 2021-09-24

**Authors:** Raquel Martinho, Gabriela Correia, Rafaela Seixas, Ana Oliveira, Soraia Silva, Paula Serrão, Carlos Fernandes-Lopes, Cristina Costa, Mónica Moreira-Rodrigues

**Affiliations:** ^1^Laboratory of General Physiology, Institute of Biomedical Sciences Abel Salazar, University of Porto (ICBAS/UP), Porto, Portugal; ^2^Center for Drug Discovery and Innovative Medicines, University of Porto (MedInUP), Porto, Portugal; ^3^Department of Pharmacology and Therapeutics, Faculty of Medicine, University of Porto (FMUP), Porto, Portugal; ^4^Department of Research, BIAL, Porto, Portugal

**Keywords:** post-traumatic stress disorder, contextual traumatic memory, dopamine β-hydroxylase, noradrenaline, nepicastat

## Abstract

Post-traumatic stress disorder (PTSD) is a common anxiety mental disorder and can be manifested after exposure to a real or perceived life-threatening event. Increased noradrenaline and adrenaline in plasma and urine have been documented in PTSD. Dopamine-β-hydroxylase (DBH) catalyzes the conversion of dopamine to noradrenaline and consequently, DBH inhibition reduces catecholamines. Our aim was to evaluate if nepicastat treatment decreases PTSD signs in an animal model. Wild-type (129x1/SvJ) female mice were submitted to PTSD induction protocol. DBH-inhibitor nepicastat (30 mg/kg) or vehicle (0.2% HPMC) were administered once daily since day 0 until day 7 or 12. The percentage of freezing was calculated on days 0, 1, 2, and 7, and behavioral tests were performed. Quantification of nepicastat in plasma and DBH activity in the adrenal gland was evaluated. Catecholamines were quantified by HPLC with electrochemical detection. mRNA expression of *Npas4* and *Bdnf* in hippocampus was evaluated by qPCR.Mice in the PTSD-group and treated with nepicastat showed a decrease in freezing, and an increase in the time spent and entries in open arms in elevated plus maze test. In mice treated with nepicastat, adrenal gland DBH activity was decreased, and catecholamines were also decreased in plasma and tissues. On day 7, in mice treated with nepicastat, there was an increase of *Npas4* and *Bdnf* mRNA expression in the hippocampus.In conclusion, DBH inhibitor nepicastat has an effect consistent with a decrease in the persistence of traumatic memories and anxiety-like behavior in this PTSD mice model. The disruption of traumatic memories through interference with the formation, consolidation, retrieval, and/or expression processes may be important to decrease PTSD symptoms and signs. The increase in *Npas4* and *Bdnf* mRNA expression in the hippocampus may be important to develop a weaker traumatic contextual memory after nepicastat treatment.

## Introduction

Previous research discovered that when a stored memory is recalled, it becomes susceptible to disruption for a short period (Nader et al., 2000; Alberini, 2005). This finding suggests that it may be possible to weaken or even erase memories of traumatic experiences that have resulted in post-traumatic stress disorder (PTSD). Memories of negative emotional events tend to last a long period, frequently remaining detailed and vivid (Brown and Kulik, 1977; Kensinger et al., 2006). Memory of neutral experiences, on the other hand, tends to fade over time.

PTSD is a common anxiety disorder and may develop after exposure to exceptionally horrifying or threatening events. The persistence of memories of negative emotional events can be adaptive by influencing behavior in similar situations in the future. Nevertheless, in some cases, the persistence of negative memories can become maladaptive, as is the case of intrusive memories in PTSD, where memories of traumatic experiences continue to intrude involuntarily into consciousness, causing significant distress (American Psychiatric Association, 2013). Elucidating the mechanisms behind the recall and persistence of negative emotional memories is thus crucial for both basic cognitive science and clinical psychopathology research.

Usually, PTSD patients show symptoms of intrusion, avoidance, arousal, alterations in mood and cognition, and show deficits in the extinction of fear memory (Lissek et al., 2005; Inslicht et al., 2013). Also, these patients show an increase of stress hormones, namely catecholamines, such as noradrenaline (NA) and adrenaline (AD) in urine and plasma (Shalev et al., 1992; Sherin and Nemeroff, 2011). Besides, when exposed to trauma-related contexts they manifest greater changes in heart rate, blood pressure, and skin conductance than controls. In fact, persistent hyperactivity of the autonomic sympathetic system was detected in PTSD patients (Li et al., 2006; Sherin and Nemeroff, 2011).

Women are two to three times more likely than men to suffer from PTSD (Olff, 2017). The preponderance of PTSD in women may be due to causes not related to trauma, such as stress hormone sensitization in reaction to early adverse experiences, intrinsic neuroendocrine factors, and subjective perception of the event. In addition, there are gender disparities in rape and sexual assault rates, including greater exposure to intimate partner abuse. Women with PTSD can experience more symptoms, have a longer course of illness, and have a lower quality of life than men (Seedat et al., 2005).

To study PTSD, several animal models have been developed using different types of traumatic events (Pynoos et al., 1996; Deslauriers et al., 2018). The PTSD animal model used in this study is based on the concept that foot shock exposure will trigger the pathophysiological process and the main symptomatology of PTSD in animals, including increased contextual traumatic memory and anxiety-like behavior (Li et al., 2006; Zhang et al., 2012; Martinho et al., 2020). In this model, the administration of multiple foot shocks has been confirmed to mimic the traumatic event (Li et al., 2006; Zhang et al., 2012; Verma et al., 2016; Martinho et al., 2020). Also, contextual reminders in this animal model of PTSD seem to parallel the exposure to contextual cues present throughout an aversive stressful situation. This is expected to induce the re-experiencing of the traumatic event (Gisquet-Verrier et al., 2004), which seems to reproduce some of the features observed in PTSD patients.

We have shown in previous studies that mice deficient in AD (phenylethanolamine-*N*-methyltransferase-knockout, Pnmt-KO mice) have reduced contextual fear learning (Toth et al., 2013; Alves et al., 2016). Also, AD administered peripherally restored traumatic memories in Pnmt-KO mice (Martinho et al., 2020). In addition, catecholamines are increased in this PTSD mice model suggesting a causal role for AD in contributing to the persistence of contextual traumatic memories and anxiety-like behavior in PTSD (Martinho et al., 2020).

On the other hand, it was previously shown that dopamine-β-hydroxylase (DBH) knockout (DBH-KO) mice exhibit reduced contextual fear memory, which was restored by isoprenaline (β-adrenoceptor agonist; Murchison et al., 2004). DBH catalyzes the conversion of dopamine (DA) to NA (Rios et al., 1999) and, consequently, DBH inhibition reduces NA and AD, and increases DA (Bourdélat-Parks et al., 2005; Schroeder et al., 2010; Devoto et al., 2014; Igreja et al., 2015; Loureiro et al., 2015). Numerous DBH inhibitors have been described and reported (Ishii et al., 1975; Kruse et al., 1987; Ohlstein et al., 1987), but none had marketing approval due to poor DBH selectivity, low potency (Beliaev et al., 2009), and/or substantial adverse effects (Kruse et al., 1986). Nepicastat is a highly potent central and peripheral DBH inhibitor that, in dogs (Stanley et al., 1997) and rats (Bonifácio et al., 2015; Loureiro et al., 2015), produced a dose-dependent reduction in NA in peripheral and central tissues. Therefore, it is effective in modulating the sympathetic nervous system, which may be useful in diseases with sympathetic hyperactivity, such as PTSD.

DBH inhibition causes a gradual sympathetic slowdown by contrast to acute sympathetic inhibition triggered by β-adrenoceptor antagonists, thus reducing the negative hemodynamic impacts of the latter (Hegde and Friday, 1998). To our knowledge, there are no described studies with nepicastat treatment in PTSD mice models. The aim of the present study was to evaluate if inhibition of DBH by treatment with DBH-inhibitor nepicastat interferes with the recall and persistence of traumatic memories. This approach could be a potential new therapeutic strategy for PTSD treatment. In this study, we will induce a PTSD mice model and treat the animals daily with vehicle or nepicastat until day 12 and evaluate traumatic contextual memory and anxiety-like behavior, DBH activity in adrenal gland, catecholamines levels in plasma and tissues, and mRNA expression of hippocampal relevant genes in contextual fear memory.

## Material and Methods

### Animals

All animal care and experimental protocols were carried out in accordance with European Directive number 63/2010/EU, transposed to Portuguese legislation by Directive Law 113/2013 and 1/2019, and approved by the Organism Responsible for Animal Welfare in Faculty of Medicine of University of Porto and National Authority for Animal Health (DGAV). Adult female mice (8–12 weeks old; 129x1/SvJ; *n* = 28) were kept under controlled environmental conditions (12 h light/dark cycle, room temperature 23 ± 1°C, humidity 50%, autoclaved drinking water, mice diet (4RF21/A); Mucedola, Milan, Italy). Animals were group-housed and experiments were performed in the light phase. The light phase started from 8 a.m. and 8 p.m. and the behavioral testing and physiological measurements were performed between 9 a.m. and 1 p.m. Between two and five mice were living in a cage and they were fed *ad libitum*. It was previously described that female rodents placed in groups synchronize their ovarian cycles (McClintock, 1978).

### PTSD Mice Model

**Figure 1:**
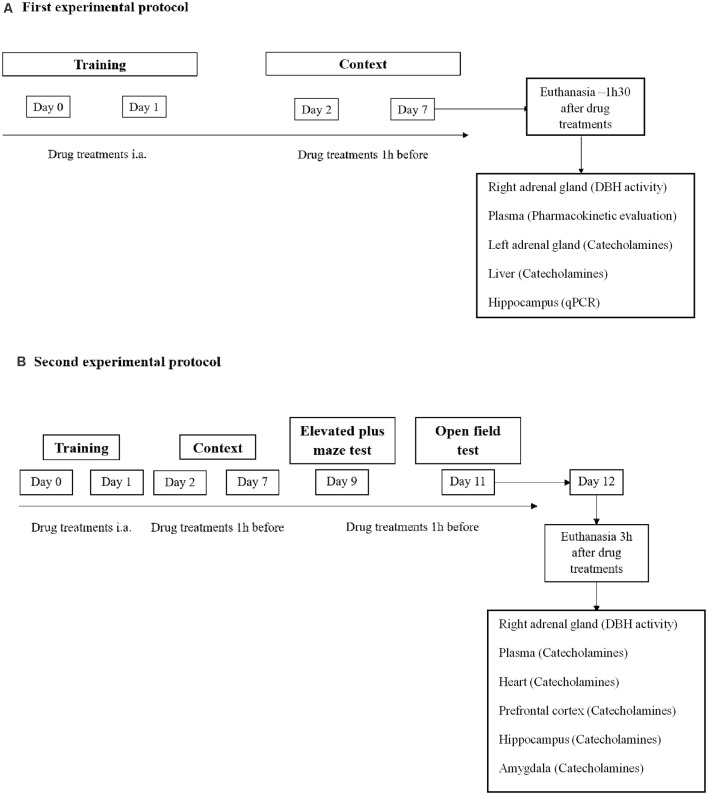
Schematic representation of the experimental design: treatments, behavioral protocols, and samples collection. **(A)** First experimental protocol and **(B)** second experimental protocol. i.a., immediately after.

PTSD mice model was performed as previously described (Li et al., 2006; Zhang et al., 2012; Verma et al., 2016; Martinho et al., 2020). In the two experimental protocols (Figures 1A,B), mice were exposed to an aversive procedure consisting of two training sessions (days 0 and 1). A clear Plexiglass chamber with a metal grid floor wired to a stimulus generator was used for the training sessions. On both days, the mice had a 2-min habituation period and were then submitted to 15 electric shocks (intensity, 0.8 mA; duration, 10 s; interval between sessions, 10 s), during a total time of 5 min. After the training session, the mice were re-exposed on days 2 and 7 to the aversive context. Re-exposure consisted in introducing the mice to the same conditioned chamber without applying foot shocks for 8 min. Freezing was defined as the absence of movement except for respiration for at least 3 s (Valentinuzzi et al., 1998). In the clear Plexiglass chamber used, a timer is placed on the top of the chamber to be able to evaluate freezing time using a camera and video software. The freezing time was manually scored and monitored when freezing behavior lasts for at least 3 s. Vocalization response was defined as the audible vocalization in response to the shock. We considered audible vocalization when after footshocks were given to mice, these animals emitted a high-pitched squeak. We measured the number of times the animal vocalized during the procedure. Jump response was defined as the removal of at least three paws from the grid floor (Rocinholi et al., 1997). The mice’s behavior was recorded with a digital video camera Sony HDR-CX405 (Sony Corporation, Japan). All quantifications were performed manually and blinded (Figure 1).

### Drug Treatments

In the first and second experimental protocol, mice were administered orally (p.o.) with nepicastat (30 mg/kg; dissolved in 0.2% HPMC; *n* = 15) or vehicle (0.2% HPMC; *n* = 13) once a day from days 0–7 (Figure 1A) or once a day from days 0–12 (Figure 1B). On training sessions days, nepicastat or vehicle were administered immediately after each session. On days 2, 7, 9, and 11 nepicastat or vehicle were administered 1 h before the behavioral test. On the other days when no tests were performed, nepicastat was administered between 9 a.m. and 10 a.m. Tissue samples were collected in day 7 of the first experimental protocol approximately 1 h 30 min after drug treatments (Figure 1A) or in day 12 approximately 3 h after nepicastat or vehicle administration of the second protocol (Figure 1B). The timeline of the experimental design, behavioral protocols, treatments, and samples collection is presented in Figure 1. Nepicastat hydrochloride was provided by BIAL-Portela and Cª, S.A. (S. Mamede Coronado, Portugal) and was synthesized in BIAL’s Chemical Research Laboratory with purities above 95%.

### Behavioral Tests

#### Elevated Plus Maze Test

Nine days after PTSD induction the elevated plus maze test was conducted (Figure 1), as previously described (Pellow et al., 1985; Martinho et al., 2020). The apparatus consisted of two open arms (40 × 10 cm) alternating at right angles with two arms enclosed by 20 cm high walls. The four arms delimited a central area of 5 cm^2^. The whole apparatus was placed 60 cm above the floor. The test began by placing the animal in the center with its head facing a closed arm. The time spent in open arms, open arm entries, and total arm entries during 5 min were recorded with a digital video camera Sony HDR-CX405 (Sony Corporation, Japan) and analyzed manually and blinded, and a four paws criterion was used for arm entries (Li et al., 2006; Zhang et al., 2012).

#### Open Field Test

Eleven days after PTSD induction the open field test was conducted (Figure 1), as previously described (Pynoos et al., 1996; Mitra et al., 2016; Martinho et al., 2020). The open field wooden chamber (50 × 50 × 30 cm) had black lines on the floor delineating twelve peripheral squares (12.5 × 12.5 cm) and a central square (25 × 25 cm). Each animal was placed in the corner of the arena and the number of squares crossed, entries in the center, and feces were recorded for 10 min with a digital video camera Sony HDR-CX405 (Sony Corporation, Japan) and analyzed manually and blinded. The total distance traveled was recorded and analyzed using ToxTrac ver 2.84^1^ (Rodriguez et al., 2017, 2018; Henry et al., 2019).

### Quantification of Nepicastat in Plasma Samples

Mice were anesthetized (ketamine, 100 mg/kg and xylazine, 10 mg/kg; i.p.) and blood was collected from the left ventricle to heparinized tubes approximately 1h30 min after the last administration of nepicastat (30 mg/kg) and after contextual behavior evaluation on day 7 (Figure 1A). After collection, blood samples were centrifuged at 1,500× *g*, for 10 min, at 4°C. The resulting plasma was stored at −80°C. Plasma nepicastat concentration was quantified by liquid chromatography coupled to tandem mass spectrometry (LC-MS/MS; 6460, Triple Quad LC-MS Agilent Technologies, USA) by BIAL-Portela and Cª, S.A. (S. Mamede Coronado, Portugal), as previously described (Loureiro et al., 2013; Pires et al., 2015). The limit of quantification was 50 ng/ml.

### Dopamine-β-Hydroxylase (DBH) Activity Determination

Right adrenal gland (AG) samples were collected approximately 1 h 30 min (day 7) and 3 h (day 12) after the last administration of nepicastat (30 mg/kg) or vehicle (0.2% HPMC; Figure 1), submerged in Tris-HCl (50 mM; pH 7.4) and kept at −80 °C. DBH activity was measured by BIAL-Portela and Cª, S.A., as previously described (Loureiro et al., 2014).

### Quantification of Catecholamines

Seven and 12 days after PTSD induction mice were anesthetized (ketamine, 100 mg/kg and xylazine, 10 mg/kg; i.p.), the left adrenal gland was collected on day 7 and submerged in perchloric acid (PCA) 0.2 M overnight, at 4°C, and frozen at −80°C. Also, the liver was collected on day 7 (Figure 1A), and the heart, prefrontal cortex, amygdala, and hippocampus were collected on day 12 (Figure 1B) submerged in perchloric acid (PCA) 0.2 M overnight, at 4 °C, and frozen at −80 °C. The brain dissection was as follows. The cerebellum and the pons were separated from the brain by a coronal incision in the transverse fissure. An incision was made between the olfactory bulb and the frontal cortex, and between the frontal cortex and the beginning of the corpus callosum. Then the hippocampus was gently separated from the cortex and collected (Chiu et al., 2007). Finally, the amygdala was identified ventrally to the temporal lobe and separated from the cortex.

Twelve days after PTSD induction, blood was collected by left ventricle puncture to a heparinized tube and the samples were centrifuged and frozen at −80°C. The catecholamines present in plasma, liver, heart, prefrontal cortex, hippocampus, and amygdala were concentrated by alumina method, as previously described (Moreira-Rodrigues et al., 2014). Briefly, 50 mg of alumina and 20 μl (100 ng/ml), or 50 μl of 3,4-Dihydroxybenzylamine (DHBA, 500 ng/ml), respectively, were added to plasma or tissue samples (liver, heart, and hippocampus). After adjustment to pH of 8.3–8.6, with Tris-EDTA (1.5 M, pH = 8.6), the samples were shaken using an autonomic shaker (Analogue Orbital Shaker 3005, GFL) for 15 min at room temperature and maximum frequency. Subsequently, alumina was rested and after two successive washes with bi-distilled water at 4°C, 500 μl of bi-distilled water at 4°C were added. The alumina was then centrifuged at 1,250× *g*, for 2 min at 4°C, in a tube with a filter. The filter with the alumina and 200 μl of PCA (0.2 M) was added to a new tube and centrifuged at 1,250× *g*, for 2 min at 4°C. For catecholamines quantification in the adrenal gland, the PCA of each sample was transferred to tubes with filters and centrifuged at 1,250× *g*, for 2 min at 4°C. Fifty microliter of the left adrenal gland, plasma, liver, heart, and hippocampus samples were injected and separated by reverse-phase high-performance liquid chromatography (HPLC) and quantified by electrochemical detection. The results of catecholamines were expressed in nmol/AG for the adrenal gland, pmol/ml for plasma, and pmol/mg for liver, heart, amygdala, and hippocampus, after normalization for DHBA.

### RNA Isolation and Relative Quantification of mRNA Expression

Real-time PCR (qPCR) was performed in hippocampus samples collected on day 7 of PTSD induction (Figure 1), as previously described (Moreira-Rodrigues et al., 2007; Mendes et al., 2018; Oliveira et al., 2018; Martinho et al., 2020). Total RNA isolation was carried out with the illustra™ Isolate II RNA Mini Kit (Bioline, London, UK). The concentration and purity of the isolated RNA were measured using the NanoDrop 2000 spectrophotometer (Thermo Scientific, Waltham, MA, USA). Reverse transcription was performed in a T100™ Thermal Cycler (Bio-Rad, Hercules, CA, USA) using a Reverse Transcription kit (NZY First-Strand cDNA Synthesis Kit NZYTech-Genes and Enzymes, Lisbon, Portugal). qPCR reactions were carried out in StepOne™ real-time PCR System (Applied BioSystems, Waltham, MA, USA). Gene-specific primers (10 μM), Maxima SYBR Green qPCR Master Mix (Thermo Scientific, Waltham, MA, USA), RNase-free H_2_O (Bioline, London, UK) were mixed and cDNA was added (1:20). Instead of cDNA, RNase-free H_2_O (Bioline, London, UK) was added as a negative control. Gene-specific primers are in Table 1. Results of mRNA quantification are expressed in an arbitrary unit (AU) after normalization for Glyceraldehyde 3-phosphate dehydrogenase (GAPDH).

**Table 1:** Primers used in gene expression analysis.

Gene	Primer (5′→ 3′)
*Npas4*	F: AGCATTCCAGGCTCATCTGAA
	R: GGCGAAGTAAGTCTTGGTAGGATT
*Bdnf*	F: GGACATATCCATGACCAGAAAGAAA
	R: GCAACAAACCACAACATTATCGAG
*Gapdh*	F: CCATCACCATCTTCCAGGAG
	R: GCATGGACTGTGGTCATGAG

*Npas4, neuronal PAS domain protein 4; *Bdnf*, brain-derived neurotrophic factor; *Gapdh*, Glyceraldehyde 3-phosphate dehydrogenase; F, forward primer; R, reverse primer*.

### Other Drugs

Hydroxypropyl methylcellulose, (-)-adrenaline, L-(-)-noradrenaline, dopamine hydrochloride, 2, 3-dihydroxybenzoic acid, and perchloric acid were purchased from Sigma-Aldrich (St. Louis, USA). Ketamine (Imalgene 1000, Merial, Lisboa, Portugal) and xylazine (Rompum 2%, Bayer, Lisboa, Portugal) were purchased from Agrofauna (Gaia, Portugal).

### Statistics

We used an online Sample Size Calculator^2^ to determine the minimum number of subjects that needed to be enrolled in the experiments of this study. All results were presented as means ± standard error of the means (SEM). GraphPad Prism 6 (GraphPad Software Inc., La Jolla, CA, USA) was used for all statistical analyses. Freezing behavior results were analyzed by Two-Way Analysis of Variance (ANOVA) repeated measures followed by Sidak’s *post hoc* test using treatment as “between-subjects factor” and time as “within-subjects factor” (repeated measure). Results regarding jump, vocalization, elevated plus maze test, open field test, DBH activity, catecholamines concentration, and qPCR were analyzed by Student’s *t*-test. Cohen’s *d* effect sizes were calculated for Student’s *t*-tests and partial eta squared ($\eta_{\text{p}}^{2}$) effect sizes were calculated for ANOVAs. We also evaluated the presence of outliers using GraphPad Prism 6. For all analyses, significance level was set at 0.05.

## Results

### Nepicastat Treatment Decreases Contextual Fear Memory in PTSD Mice Model

**Figure 2:**
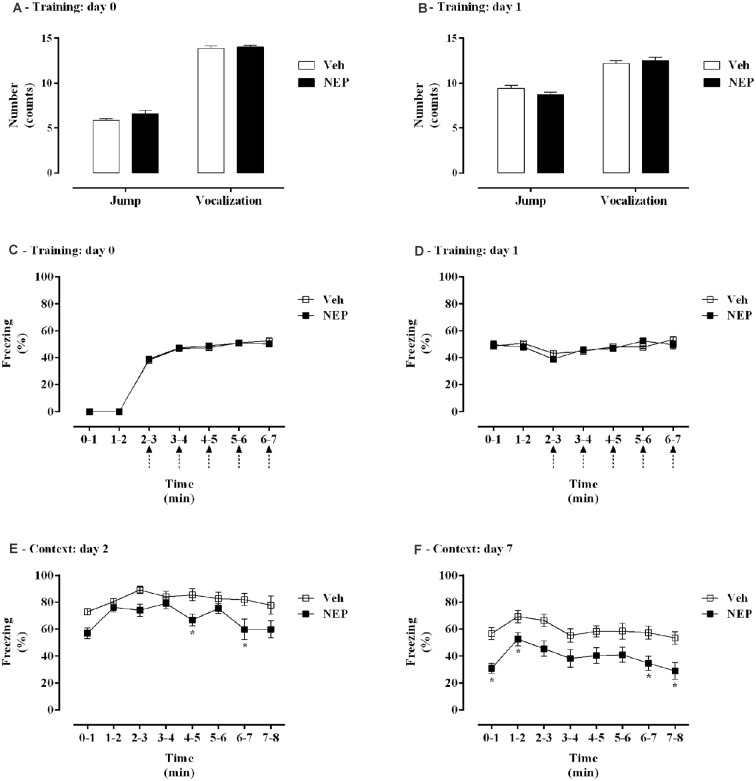
**(A,B)** Shock responsivity and **(C–F)** freezing behavior during induction of post-traumatic stress disorder (PTSD) on **(A,C)** day 0, **(B,D)** day 1, **(E)** day 2, and **(F)** day 7. Values are means ± SEM of 13–15 mice per group from both experimental protocols. Veh, mice in the PTSD-group and treated with vehicle; NEP, mice in the PTSD-group and treated with nepicastat; ↑ = 3 footshocks delivered with a duration of 10 s and a 10 s interval; *, significantly different from correspondent values in mice in the PTSD-group and treated with vehicle (*p* < 0.05).

The effects of nepicastat treatment in freezing behavior of PTSD animals were assessed and are shown in Figure 2. During training days 0 and 1, no differences were observed in jump (*t*_(26)_= 1.05, *p* = 0.3041; Cohen’s *d* = 0.41; Figure 2A; *t*_(26)_ = 1.79, *p* = 0.085; Cohen’s *d* = 0.68; Figure 2B), vocalization (*t*_(26)_= 0.021, *p* = 0.9835; Cohen’s *d* = 0.0076; Figure 2A; *t*_(26)_ = 0.64, *p* = 0.5261; Cohen’s *d* = 0.25; Figure 2B), or freezing responses (treatment: *F*_(1,26)_ = 0.0001832, *p* = 0.9893, $\eta_{\text{p}}^{2}$ = 0.000007, Figure 2C; treatment: *F*_(1,26)_ = 0.2999, *p* = 0.5886, $\eta_{\text{p}}^{2}$ = 0.01, Figure 2D) between groups. Moreover, on days 2 and 7 mice in the PTSD-group and treated with nepicastat showed a significant decrease in freezing behavior compared to mice treated with vehicle (Figures 2E,F). A significant effect of time (*F*_(7,182)_ = 5.14, *p* < 0.0001, $\eta_{\text{p}}^{2}$ = 0.16; Figure 2E; *F*_(7,238)_ = 5.31, *p* < 0.0001, $\eta_{\text{p}}^{2}$ = 0.13; Figure 2F) and treatment (*F*_(1,26)_ = 10.49, *p* = 0.0033, $\eta_{\text{p}}^{2}$ = 0.29; Figure 2E; *F*_(1,34)_ = 15.93, *p* = 0.0003, $\eta_{\text{p}}^{2}$ = 0.32; Figure 2F) was observed.

### Nepicastat Treatment Decreases Anxiety-Like Behavior in PTSD

**Figure 3:**
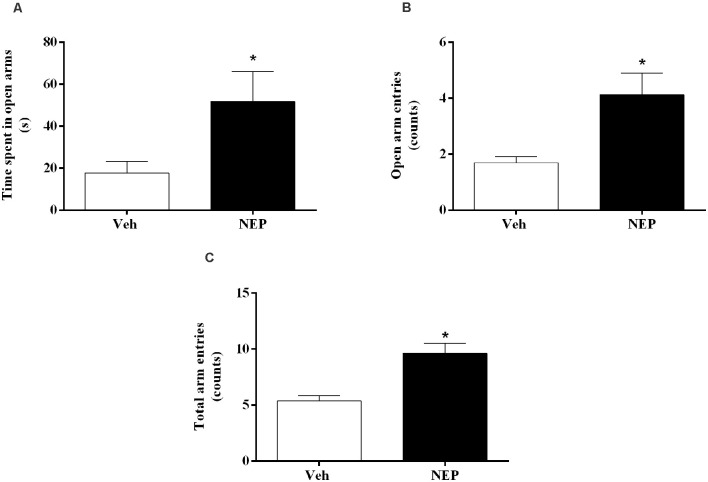
**(A)** Time spent in open arms, **(B)** open arm entries, and **(C)** total arm entries of the elevated plus maze test, on day 9 after post-traumatic stress disorder (PTSD) induction. Values are means ± SEM of eight mice per group. Veh, mice in the PTSD-group and treated with vehicle; NEP, mice in the PTSD-group and treated with nepicastat; *, significantly different from correspondent values in mice in the PTSD-group and treated with vehicle (*p* < 0.05).

Nine days after PTSD induction the elevated plus maze test was performed to assess the effects of nepicastat treatment on anxiety-like behavior. In this test, the time spent in open arms (*t*_(22)_ = 2.68, *p* = 0.0141, Cohen’s *d* = 1.06; Figure 3A), open arm entries (*t*_(22)_ = 3.97, *p* = 0.0007, Cohen’s *d* = 1.48; Figure 3B), and the total number of arm entries (*t*_(22)_ = 4.61, *p* = 0.0001, Cohen’s *d* = 1.90; Figure 3C) were significantly increased in mice in the PTSD-group and treated with nepicastat when compared to mice treated with vehicle.

### Nepicastat Treatment Did Not Affect Spontaneous Locomotor Activity

**Figure 4:**
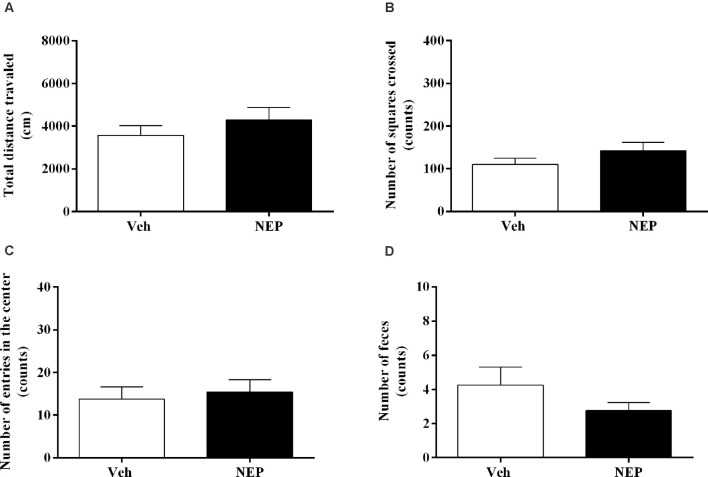
**(A)** Total distance traveled and number of **(B)** squares crossed, **(C)** entries in the center, and **(D)** feces of the open field test, on day 11 after post-traumatic stress disorder (PTSD) induction. Values are means ± SEM of eight mice per group. Veh, mice in the PTSD-group and treated with vehicle; NEP, mice in the PTSD-group and treated with nepicastat.

Eleven days after PTSD induction the open field test was performed to assess the effects of nepicastat treatment on locomotor activity. There were no significant differences in the total distance traveled (*t*_(14)_ = 0.97, *p* = 0.3467, Cohen’s *d* = 0.49; Figure 4A), the number of squares crossed (*t*_(14)_ = 1.33, *p* = 0.2038, Cohen’s *d* = 0.67; Figure 4B), entries in the center (*t*_(14)_ = 0.40, *p* = 0.6987, Cohen’s *d* = 0.20; Figure 4C), and feces (*t*_(14)_ = 0.13, *p* = 0.2216, Cohen’s *d* = 0.64; Figure 4D) between groups of animals. Detailed point of estimates of Figure 4 are in Supplementary Table 1.

### Accurate Concentration of Nepicastat in Plasma Samples

To assess the effective exposure of nepicastat in administered animals, the levels of the compound were quantified in plasma. Seven days after PTSD induction and daily treatment with nepicastat, the mean concentration of nepicastat in plasma 1 h after last oral administration was 10,046 ± 767 ng/ml. Plasma nepicastat levels in mice treated with vehicle were 0.0000 ng/ml.

### Nepicastat Decreases Dopamine-β-Hydroxylase (DBH) Activity in the Adrenal Gland

**Figure 5:**
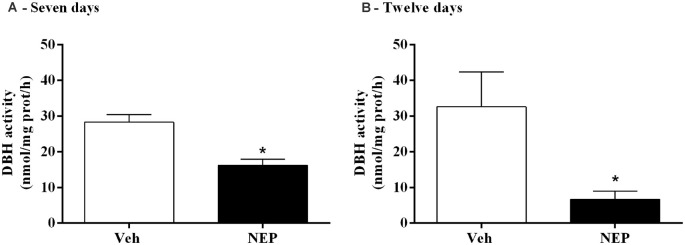
DBH activity in the adrenal gland after treatment with nepicastat **(A)** 7 and **(B)** 12 days after post-traumatic stress disorder (PTSD) induction. Values are means ± SEM of 5–8 mice per group. Veh, mice in the PTSD-group and treated with vehicle; NEP, mice in the PTSD-group and treated with nepicastat; *, significantly different from correspondent values in mice in the PTSD-group and treated with vehicle (*p* < 0.05).

To evaluate the inhibitory profile on DBH by nepicastat, DBH activity in the adrenal gland was measured. After daily treatment with nepicastat, DBH activity in the adrenal gland was significantly decreased seven (*t*_(10)_ = 4.42, *p* = 0.0013, Cohen’s *d* = 2.58; Figure 5A) and 12 days (*t*_(14)_ = 2.57, *p* = 0.0223, Cohen’s *d* = 1.28; Figure 5B) after PTSD induction compared to mice treated with vehicle.

### Nepicastat Decreases NA and AD in PTSD

**Figure 6:**
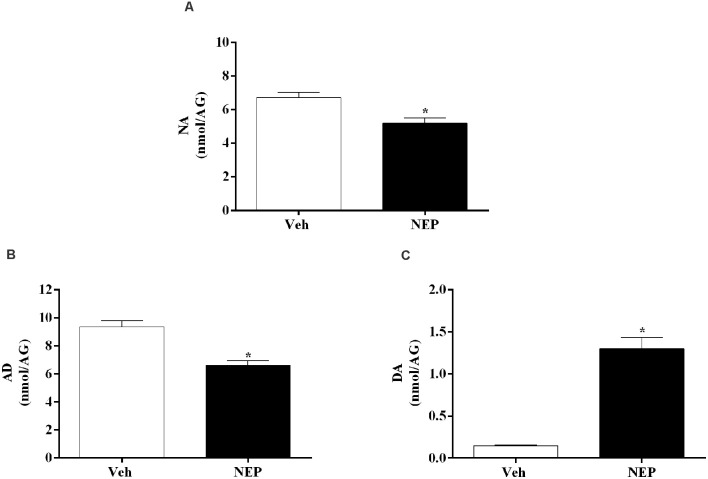
Concentration of **(A)** noradrenaline (NA), **(B)** adrenaline (AD), and **(C)** dopamine (DA) in the adrenal gland (AG) expressed as nmol/AG on day 7 after post-traumatic stress disorder (PTSD) induction. Values are means ± SEM of 5–7 mice per group. Veh, mice in the PTSD-group and treated with vehicle; NEP, mice in the PTSD-group and treated with nepicastat; *, significantly different from correspondent values in mice in the PTSD-group and treated with vehicle (*p* < 0.05).

To assess the effects of nepicastat treatment in catecholamines, catecholamine levels were quantified in plasma and tissues. Seven days after PTSD induction, NA (*t*_(10)_ = 3.42, *p* = 0.0065, Cohen’s *d* = 2.03; Figure 6A) and AD (*t*_(10)_ = 5.46, *p* = 0.0003, Cohen’s *d* = 3.13; Figure 6B) in the adrenal gland were decreased whereas DA (*t*_(10)_ = 6.99, *p* < 0.0001, Cohen’s *d* = 4.48; Figure 6C) was increased in mice in the PTSD-group and treated with nepicastat compared to mice treated with vehicle.

**Figure 7:**
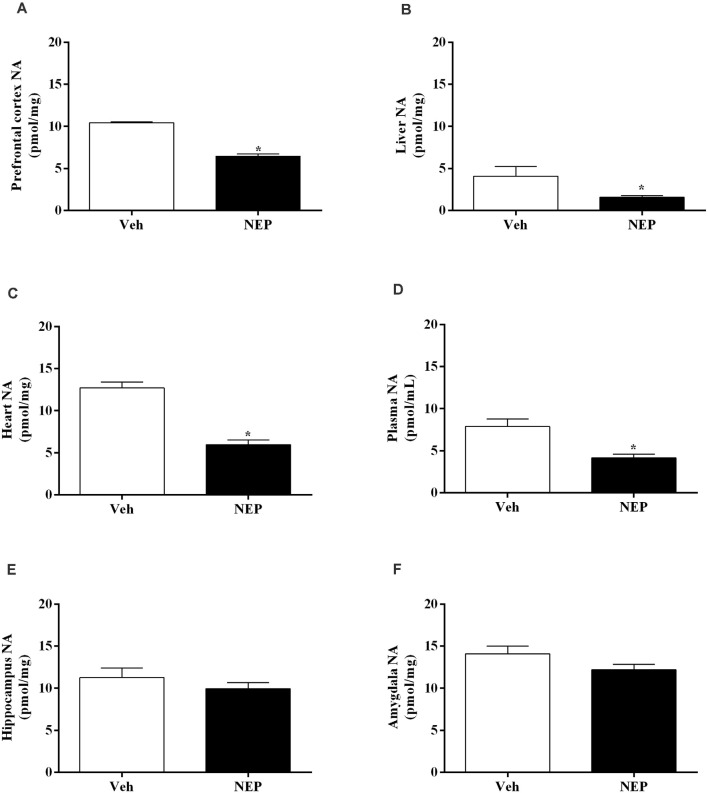
Noradrenaline (NA) concentration in the **(A)** prefrontal cortex, **(B)** liver, **(C)** heart, **(D)** plasma, **(E)** hippocampus, and **(F)** amygdala in mice induced with post-traumatic stress disorder (PTSD). Values are means ± SEM of eight mice per group. Veh, mice in the PTSD-group and treated with vehicle; NEP, mice in the PTSD-group and treated with nepicastat; *, significantly different from correspondent values in mice in the PTSD-group and treated with vehicle (*p* < 0.05).

Moreover, NA decreased in the prefrontal cortex (*t*_(10)_ = 11.79, *p* < 0.0001, Cohen’s *d* = 7.39, Figure 7A), liver (*t*_(10)_ = 2.55, *p* = 0.0288, Cohen’s *d* = 1.35; Figure 7B), heart (*t*_(14)_ = 7.64, *p* < 0.0001, Cohen’s *d* = 3.82; Figure 7C), and plasma (*t*_(14)_ = 3.69, *p* = 0.0024, Cohen’s *d* = 1.84; Figure 7D) of mice in the PTSD-group and treated with nepicastat compared to mice treated with vehicle. No significant differences were observed in NA in the hippocampus (*t*_(14)_ = 0.98, *p* = 0.3479, Cohen’s *d* = 0.49; Figure 7E) and in the amygdala (*t*_(14)_ = 1.765, *p* = 0.1010, Cohen’s *d* = 0.90; Figure 7F) between groups of animals.

### Nepicastat Decreases *Npas4* and *Bdnf* Gene Expression in the Hippocampus

**Figure 8:**
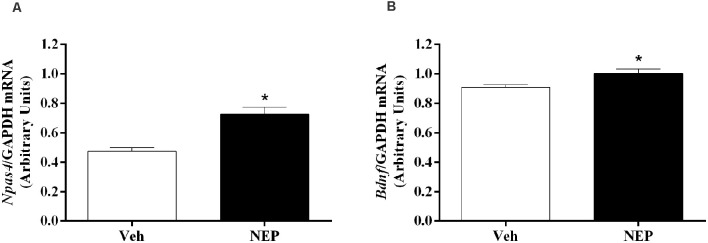
Hippocampus mRNA expression of **(A)** *Npas4* and **(B)** *Bdnf* on day 7 of post-traumatic stress disorder (PTSD) induction. Values are means ± SEM of 5–7 mice per group. Results of mRNA are expressed as arbitrary units (AUs) after normalization for Glyceraldehyde 3-phosphate dehydrogenase (GAPDH). Veh, mice in the PTSD-group and treated with vehicle; NEP, mice in the PTSD-group and treated with nepicastat; *, significantly different from correspondent values in mice in the PTSD-group and treated with vehicle (*p* < 0.05).

Hippocampus mRNA expression of neuronal PAS domain protein (*Npas4, t*_(10)_ = 3.77, *p* = 0.0044; Cohen’s *d* = 2.63; Figure 8A) and brain-derived neurotrophic factor (*Bdnf, t*_(10)_ = 2.33, *p* = 0.0450; Cohen’s *d* = 1.61; Figure 8B) were significantly increased in mice in the PTSD-group and treated with nepicastat when compared to mice treated with vehicle.

## Discussion

After exposure to a traumatic event, PTSD is more likely to develop in females than in males since estrogen promotes catecholamine production (Breslau et al., 1999; McDermott et al., 2015). In fact, sex may be critical concerning the pathogenesis of anxiety disorders and its treatments (Milad et al., 2006). Thus, considering the existence of sex dimorphism in brain morphology and neurotransmission (de Vries and Södersten, 2009; de Lima Xavier et al., 2019), it would be interesting to pay more attention to PTSD treatments in women, and therefore this study is with female mice.

A validated mice model of PTSD was used in this study (Li et al., 2006; Zhang et al., 2012; Verma et al., 2016; Martinho et al., 2020). The absence of differences in vocalization, jump, and freezing responses in both days 0 and 1 (first and second training days of PTSD mice model, respectively) suggests that pain perception of the foot shocks was not different between groups. Contextual reminders in this PTSD animal model may induce the re-experiencing of the traumatic event, which may be analogous to what is experienced by PTSD patients (Gisquet-Verrier et al., 2004). A measure of conditioned associative fear memory is the freezing response upon re-exposure to the contextual reminders, reflecting the response to trauma-related cues as a PTSD symptom (Siegmund and Wotjak, 2007).

We have previously shown that AD may contribute to the persistence of contextual traumatic memories in this PTSD mice model (Martinho et al., 2020). Following a stressful event, an increase in catecholamines in the bloodstream can result in the activation of the β-adrenoceptors of liver cells and the subsequent breakdown of glycogen stores, increasing blood glucose levels (Sutherland et al., 1968; Gray et al., 1980; Dufour et al., 2009). The released glucose crosses the blood-brain barrier and may improve hippocampal-dependent contextual learning (Gold, 2014). In a stressful environment, this increase in glucose may lead to the synthesis of neuromodulators, which will activate signalling pathways that can influence the process of learning and memory consolidation (Durkin et al., 1992; Gold, 2014). Therefore, in the central nervous system, glucose may be a mediator of catecholamines by providing additional energy for specific memory mechanisms, such as contextual fear learning, and in long-term memory formation be a critical component of fear memory modulation (Durkin et al., 1992; Pych et al., 2005; Alves et al., 2016; Oliveira et al., 2018).

DBH catalyzes the conversion of DA to NA and, consequently, DBH inhibition reduces NA and AD, and increases DA (Bourdélat-Parks et al., 2005; Schroeder et al., 2010; Devoto et al., 2014; Igreja et al., 2015; Loureiro et al., 2015). Nepicastat is a highly potent reversible DBH inhibitor. It has the potential to cause a gradual sympathetic slowdown (Stanley et al., 1997; Bonifácio et al., 2015; Loureiro et al., 2015), which may be useful in diseases with sympathetic hyperactivity, such as PTSD. This is the first study to report the effect of nepicastat in modulating the sympathetic nervous system in a PTSD mice model. Since nepicastat exhibits high oral absorption and distribution (Loureiro et al., 2015) it was orally administered. In our study, nepicastat plasma concentration was in agreement with previous reports (Bonifácio et al., 2015; Loureiro et al., 2015), and is consistent with an unbound plasma drug concentration that is pharmacologically active (Braggio et al., 2010). The dosage of 30 mg/kg used leads to maximal adrenal gland DBH enzyme inhibition between 1 and 8 h post-administration (Bonifácio et al., 2015; Loureiro et al., 2015; Catelas et al., 2020). This high inhibition of DBH activity allows to effectively elicit pharmacological effects, namely a gradual decrease in catecholamine levels in the following 24 h (Bonifácio et al., 2015; Loureiro et al., 2015), and therefore nepicastat was administered once daily. This dosage was already used in several studies, namely in hypertension (Stanley et al., 1997; Sabbah et al., 2000; Manvich et al., 2013; Bonifácio et al., 2015; Devoto et al., 2015; Loureiro et al., 2015; Catelas et al., 2020). Since we performed nepicastat treatment daily, in the first protocol from days 0–7 and in the second protocol from days 0–12, this administration was chronic. We cannot rule out the possibility that the acute administration of nepicastat 1 h before the behavioral tests would be sufficient to trigger the observed effects since acute administration was not evaluated. However, since catecholamine levels in the adrenal gland only decrease 4 h after acute administration (Catelas et al., 2020) it is unlikely that the acute administration could trigger the observed effects. Our study is in agreement with previous reports (Bonifácio et al., 2015; Loureiro et al., 2015; Catelas et al., 2020) and shows that nepicastat successfully inhibited adrenal gland DBH activity in this PTSD mice model, which was translated into the observed decrease in catecholamine levels. In fact, nepicastat treatment reduced AD and NA in the adrenal gland, and NA in the liver, plasma, and heart in PTSD-group mice.

In the present work, the observed traumatic contextual memory, anxiety-like behavior, and catecholamines in vehicle-treated PTSD mice were similar to our previous publication in which we compared PTSD and control mice (Martinho et al., 2020). In addition, a significant decrease in freezing behavior was observed in mice treated with nepicastat compared to mice treated with vehicle on both days of re-exposure to contextual reminders (days 2 and 7 of PTSD mice model). Thus, in this way, nepicastat treatment by decreasing catecholamines may have blunted the increase of glucose which is known to result from stressful events (Sutherland et al., 1968), and therefore decrease the recall and persistence of contextual traumatic memories in this PTSD mice model. We administered nepicastat immediately after traumatic fear learning to decrease the catecholamines surge elicited by the trauma, which may influence the traumatic memory formation and consolidation processes, and also 1 h before contextual tests to maintain a decreased catecholamines content on these days, which may influence the traumatic memory retrieval and expression processes. Therefore, nepicastat may weaken the formation, consolidation, retrieval, and/or expression processes of traumatic contextual memory. We cannot exclude that auditory cue-induced traumatic memory has a role in nepicastat treatment in PTSD since this cue-induced traumatic memory was not evaluated.

The elevated plus maze is a test based on the natural tendency of animals to avoid elevated and open places, as well as on their natural exploratory behavior in novel environments (Zhang et al., 2012). This test is a method for assessing anxiety-like responses in rodents (Pellow et al., 1985). Our results showed an increase in the time spent and the number of entries in the open arms and total arm entries in mice in the PTSD-group and treated with nepicastat compared to mice treated with vehicle. Thus, this suggests that nepicastat treatment decreases the anxiety-like behavior in mice in the PTSD-group. Since traumatic contextual memory has been described to contribute to the persistence of PTSD symptoms and signs, weakening traumatic memory with nepicastat may, therefore, play a role in decreasing PTSD symptoms and signs.

Previous studies showed that foot shocks related to situational reminders did not affect the locomotor activity of mice in an open field test performed 3–6 weeks (Pynoos et al., 1996) or 10 days (Martinho et al., 2020) after the PTSD mice model induction. In agreement, our results showed no differences between groups in the total distance traveled, number of squares crossed, and entries in the center of the open field test. Also, total arm entries in the elevated plus maze may not be an optimal measure of locomotor activity (Walf and Frye, 2007). In fact, the observed increase in total arm entries induced by nepicastat treatment was not associated with a similar effect on locomotor activity, as revealed in the open field test. Therefore, we suggest that the behavioral changes observed with nepicastat treatment are not due to differences in locomotor activity.

Nepicastat reduced NA levels in the prefrontal cortex. However, there were no changes in catecholamine levels in hippocampus and amygdala in mice induced with PTSD. Thus, nepicastat possibly showed preferential distribution to the prefrontal cortex than to hippocampus and amygdala. Taken all together, these results are in agreement with a previous study that described central and peripheral catecholamine modulation by nepicastat in spontaneously hypertensive rats and dogs (Stanley et al., 1997). The prefrontal cortex seems to be important in memory retrieval and consolidation. NA release in the prefrontal cortex operates in an inverted-U shape (Arnsten, 2007; Arnsten et al., 2015). If moderate levels of NA are released, there is preferential activation of α_2_-adrenoceptors which enhance prefrontal cortex function (Li and Mei, 1994). However, when higher levels of NA are released occurs the activation of the α_1_-adrenoceptors (Arnsten, 2000) that impairs prefrontal cortex function which is associated with symptomatic PTSD (Shin et al., 2006). Since we found a decrease of NA in the prefrontal cortex after nepicastat treatment compared to vehicle-treated PTSD mice, the DBH inhibitor nepicastat may reduce NA levels in prefrontal cortex contributing to prefrontal cortex noradrenergic hyporesponsiveness and decrease PTSD traumatic contextual memories and anxiety-like behavior.

The hippocampus is involved in contextual fear conditioning (Rudy et al., 2004). Contextual stimuli are processed in the hippocampus and the hippocampal afferents to the amygdala synapse primarily on basal nuclei. Indeed, selective neurotoxic bilateral damage to the basal nuclei disrupted contextual, but not auditory fear conditioning (Onishi and Xavier, 2010). Since the hippocampus is involved in contextual fear conditioning (Rudy et al., 2004) and catecholamines in this brain area were seen to be unaffected by nepicastat treatment, we decided to explore possible molecular mechanisms in the hippocampus underlying the observed decrease in contextual traumatic memory.

The weakening of the formation, consolidation, retrieval, and/or expression processes of traumatic contextual memory by nepicastat lead to an increase in *Npas4* mRNA expression in the hippocampus on day 7 of PTSD induction. *Npas4* is a neuronal immediate early gene and a transcription factor highly expressed in the adult hippocampus (Lin et al., 2008; Ploski et al., 2011). In previous studies, it has been suggested that higher hippocampal *Npas4* mRNA expression was related to higher activation of the hippocampus (Drouet et al., 2018), and this was shown to be consistent with a decrease in fear memory (Deschaux et al., 2015). In the hippocampal CA3 region, *Npas4* may regulate a gene induction program essential for contextual fear memory (Ramamoorthi et al., 2011). Also, it has been shown that *Npas4* controls a transcriptional program that includes *Bdnf* gene (Lin et al., 2008). *Bdnf* mRNA expression also increases in the hippocampus on day 7 of PTSD mice model after treatment with nepicastat. BDNF is a neurotrophin implicated in neuronal survival (Barde et al., 1982; Lewin and Barde, 1996) and in neuronal maturation (Lindsay et al., 1994), which may be important in weakening traumatic memory after nepicastat treatment.

The effectiveness of drugs to treat PTSD is being challenged (Ipser and Stein, 2012) and there are evidence-based suggestions that psychotherapy decreases rather than remits PTSD symptoms (Steenkamp and Litz, 2013). In a previous study, safety analyses showed that nepicastat was well-tolerated in healthy adults and no differences in adverse events were observed (De La Garza et al., 2015). Our study showed that nepicastat treatment might be effective in reducing PTSD symptoms in a PTSD mice model with increased catecholamine levels (AD and NA; Martinho et al., 2020), and thus could be an efficient treatment at least in humans with PTSD that have increased catecholamine plasma levels.

In conclusion, DBH inhibitor nepicastat has an effect consistent with a decrease in the persistence of traumatic memories and anxiety-like behavior in this PTSD mice model. The disruption of traumatic memories through interference with the formation, consolidation, retrieval, and/or expression processes may be important to decrease PTSD symptoms and signs. The increase in *Npas4* and *Bdnf* mRNA expression in the hippocampus may be important to develop a weaker traumatic contextual memory after nepicastat treatment.

## Data Availability Statement

The original contributions presented in the study are included in the article/Supplementary Materials, further inquiries can be directed to the corresponding author.

## Ethics Statement

The animal study was reviewed and approved by Organism Responsible for Animal Welfare in Faculty of Medicine of University of Porto and National Authority for Animal Health.

## Author Contributions

MM-R and RM conceived the study. RM performed most of the experiments and respective statistical analysis (sections “PTSD Mice Model”, “Drug Treatments”, “Behavioral Tests”, and “Quantification of Catecholamines”). GC (section “RNA Isolation” and ”Relative Quantification of mRNA Expression”), RS (sections “PTSD Mice Model” and ”Open Field Test”), AO (sections “PTSD Mice Model” and ”Quantification of Catecholamines”), SS (section “PTSD Mice Model”), and PS (section “Quantification of Catecholamines”) performed some experiments and respective statistical analysis. CF-L and CC [sections “Quantification of Nepicastat in Plasma Samples” and “Dopamine-β-Hydroxylase (DBH) Activity Determination”] performed some experiments. RM and MM-R reviewed the statistical analysis, interpreted results, and wrote the manuscript. All authors contributed to the article and approved the submitted version.

## Conflict of Interest

CF-L and CC are employed by BIAL—Portela & C^a^, S.A. (S. Mamede Coronado, Portugal). The remaining authors declare that the research was conducted in the absence of any commercial or financial relationships that could be construed as a potential conflict of interest.

## Publisher’s Note

All claims expressed in this article are solely those of the authors and do not necessarily represent those of their affiliated organizations, or those of the publisher, the editors and the reviewers. Any product that may be evaluated in this article, or claim that may be made by its manufacturer, is not guaranteed or endorsed by the publisher.

## Footnotes

^1^ https://sourceforge.net/projects/toxtrac/

^2^ https://clincalc.com/Stats/SampleSize.aspx
