# Supplementary material for: Treatment With Nepicastat Decreases Contextual Traumatic Memories Persistence in Post-traumatic Stress Disorder
Source: Front Mol Neurosci. 2021 Sep 24;14:745219. doi: 10.3389/fnmol.2021.745219 (PMC8498196; doi:10.3389/fnmol.2021.745219)
Supplement: Supplementary file 1 [file Table_1.docx]

Supplementary Material

## Supplementary Table

**Supplementary Table 1.** Total distance travelled, number of squares crossed, entries in the center, and feces of the open field test, on day 11 after post-traumatic stress disorder (PTSD) induction. Detailed point of estimates of Figure 4.

|  | Veh | NEP |
| --- | --- | --- |
| Total distance travelled | 3750 ± 450, *n* = 8 | 4293 ± 583, *n* = 8 |
| Number of squares crossed | 110 ± 14, *n* = 8 | 142 ± 20, *n* = 8 |
| Number of entries in the center | 14 ± 3, *n* = 8 | 15 ± 3, *n* = 8 |
| Number of feces | 4 ± 1, *n* = 8 | 2 ± 1, *n* = 8 |

Values are means ± SEM of 8 mice per group. Veh, mice in the PTSD-group and treated with vehicle; NEP, mice in the PTSD-group and treated with nepicastat.
